# Sensing of cardiolipin exposure on plasma membranes of apoptotic cells by EryA‐mCherry protein

**DOI:** 10.1111/febs.70290

**Published:** 2025-10-23

**Authors:** Luka Žeželj, Tadeja Bele, Anastasija Panevska, Gregor Bajc, Jan Kejžar, Miha Bahun, Nataša Poklar Ulrih, Valentina Levak, Matej Skočaj, Larisa Lara Popošek, Peter Veranič, Nataša Resnik, Kristina Sepčić

**Affiliations:** ^1^ Department of Biology, Biotechnical Faculty University of Ljubljana Slovenia; ^2^ Department of Food Science and Technology, Biotechnical Faculty University of Ljubljana Slovenia; ^3^ National Institute of Biology Ljubljana Slovenia; ^4^ Jožef Stefan International Postgraduate School Ljubljana Slovenia; ^5^ Institute of Cell Biology, Faculty of Medicine University of Ljubljana Slovenia

**Keywords:** aegerolysin, annexin V, apoptosis marker, cardiolipin, *Pleurotus*

## Abstract

Erylysin A (EryA), an aegerolysin protein produced by the edible king oyster mushroom (*Pleurotus eryngii*), interacts strongly with an invertebrate‐specific membrane sphingolipid ceramide phosphoethanolamine. Recently, a fluorescently fused variant of EryA was shown to bind to artificial and bacterial lipid membranes containing cardiolipin (CL). This tetra‐acylated glycerophospholipid, present in bacteria and in inner mitochondrial membranes of eukaryotic cells, was shown to be externalized to the plasma membrane surface during the process of apoptosis. In this work, we evaluated the interaction of EryA‐mCherry with CL‐containing artificial lipid vesicles and with mammalian cells undergoing apoptosis and compared its binding affinity and specificity to that of the well‐established apoptosis marker, annexin V‐FITC. Our results show that, in contrast to annexin V‐FITC, which binds several negatively charged glycerophospholipids, EryA‐mCherry specifically recognizes and binds CL in artificial membrane systems. However, this binding of EryA‐mCherry to CL‐supplemented membranes is less effective (*K*_D_ = 4.7 ± 1.6 μm) than that of annexin V‐FITC, whose binding is observed at nanomolar concentrations. Experiments using mammalian cells showed the ability of EryA‐mCherry to selectively label the membranes of apoptotic cells, binding to the same membrane regions as anti‐CL antibodies and annexin V‐FITC. Our data suggest that EryA‐mCherry might be used as a marker of early apoptosis, as well as a marker of CL in biological and artificial lipid membranes.

## Graphical abstract

An aegerolysin protein erylysin A (EryA) fused with mCherry binds to artificial lipid vesicles supplemented with cardiolipin (CL). This binding is much more specific than that of annexin V, which binds various negatively charged glycerophospholipids (PG, PS, PI). In mammalian cells, the CL exposition at the membrane surface represents an early hallmark of apoptosis. This surface‐exposed CL can be effectively sensed by EryA‐mCherry, proposing it as a marker of early apoptosis.

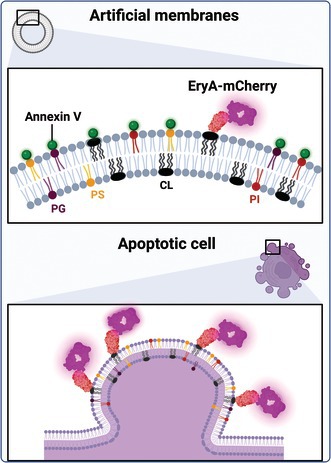

**Abbreviations**
Chol: cholesterol
CL: cardiolipin
CPE: ceramide phosphoethanolamine
DPH: diphenylhexatriene
DSA: *Datura stramonium* lectin
EGFP: enhanced green fluorescent protein
EryA: erylysin A
LUV: large unilamellar vesicle
MACPF: membrane‐attack complex/perforin
MDCK: Madin‐Darby canine kidney cells
PA: phosphatidic acid
PDI: polydispersity index
PE: phosphatidylethanolamine
PG: phosphatidylglycerol
PI: phosphatidylinositol
PlyB: pleurotolysin B
POPC: 1‐palmitoyl‐2‐oleoyl‐*sn*‐glycero‐3‐phosphocholine
PS: phosphatidylserine
RU: response unit
SM: sphingomyelin
STS: staurosporine
SUV: small unilamellar vesicle
TEM: transmission electron microscopy
TMA: trimethylammonium

## Introduction

Cardiolipin (CL) is a tetra‐acylated glycerophospholipid composed of two phosphatidic acids linked together by a glycerol bridge. The negatively charged small head group and a large hydrophobic region contribute to the conical shape of this molecule and to its strong preference for membrane regions with high negative curvature. Besides localizing at the poles and division sites in bacteria [1], CL is also found in inner mitochondrial membranes of eukaryotic cells, where it predominantly faces the membrane matrix side [2] and accounts for 25% of the total phospholipid content [3]. In mitochondria, it has a crucial role in maintaining the cristae morphology and is involved in many essential functions, including bioenergetics, the organization and optimal activity of oxidative phosphorylation complexes, as well as in their association to supercomplexes (respirasomes) [4, 5, 6, 7, 8, 9, 10, 11]. Mitochondrial CL is also involved in programmed cell death (apoptosis). During this process, CL is redistributed from the inner to the outer mitochondrial membrane, where it serves as an eat‐me signal for damaged mitochondria [12]. Cardiolipin species can also be rapidly redistributed to other cell membranes, including the plasma membrane. There, CL can be detected as early as 20 min after the induction of apoptosis, thus preceding the translocation of phosphatidylserine from the inner to the outer leaflet of the plasma membrane, which is considered a hallmark of apoptosis [13, 14, 15].

Recent studies have highlighted the association of the altered levels of CL with several pathophysiological conditions, such as Barth syndrome, cardiovascular and metabolic disorders, cancer, and neurodegeneration. These discoveries point to the emerging role of this lipid as a potential biomarker to diagnose and monitor these conditions, or even to act as a pharmacological target in treating these diseases [11, 16, 17, 18, 19, 20]. Due to its highly tissue‐specific acyl chain composition and alterations in its concentration and acylation pattern in different pathologies, CL is usually measured in plasma of these patients by advanced analytical methods such as liquid chromatography coupled with mass spectroscopy, shotgun lipidomics, ion mobility spectrometry, and radiolabeling [18]. Another cost‐effective approach to monitor the distribution and concentration of CL is the use of fluorescently labeled molecules. So far, the localization of CL in bacteria and in eukaryotic cells has been mainly monitored by a positively charged fluorescent dye 10‐nonyl acridine orange [21]. The use of this molecule, however, also has several drawbacks, such as low sensitivity and low selectivity for CL (especially over its structural analogue, phosphatidylglycerol), photo‐instability, photo‐induced cytotoxicity, and its dependence on the mitochondrial membrane potential for retention [19]. Over the past two decades, several alternative small fluorescent molecules with improved specificity have been developed as CL‐specific probes [9, 19, 22, 23, 24, 25, 26, 27, 28].

Recently, it has been shown that a 15 kDa recombinant aegerolysin‐like protein erylysin A fused with the enhanced green fluorescent protein (EryA‐EGFP) specifically interacts with artificial lipid vesicles supplemented with CL and accumulates in CL‐enriched membrane regions (e.g., in cytoplasmic leaflets of the plasma membranes at cell poles) when expressed in *Escherichia coli* [29]. This discovery has inspired us to further explore the intensity and specificity of the interaction of the fluorescently fused EryA (here in the form of EryA‐mCherry) with CL‐supplemented artificial lipid membranes. Furthermore, we aimed to evaluate the possible application of EryA‐mCherry as a marker of surface‐exposed CL in apoptotic cells, to compare its interactions with cell membranes with those of another evaluated marker of early apoptosis, annexin V‐FITC, and to correlate EryA‐mCherry labeling with anti‐CL immunolabeling. Our combined results show that EryA‐mCherry specifically recognizes and binds CL in artificial membrane systems and in plasma membranes of mammalian cells undergoing apoptosis.

## Results

### Characterization of cardiolipin‐containing artificial lipid vesicles

**Fig. 1:**
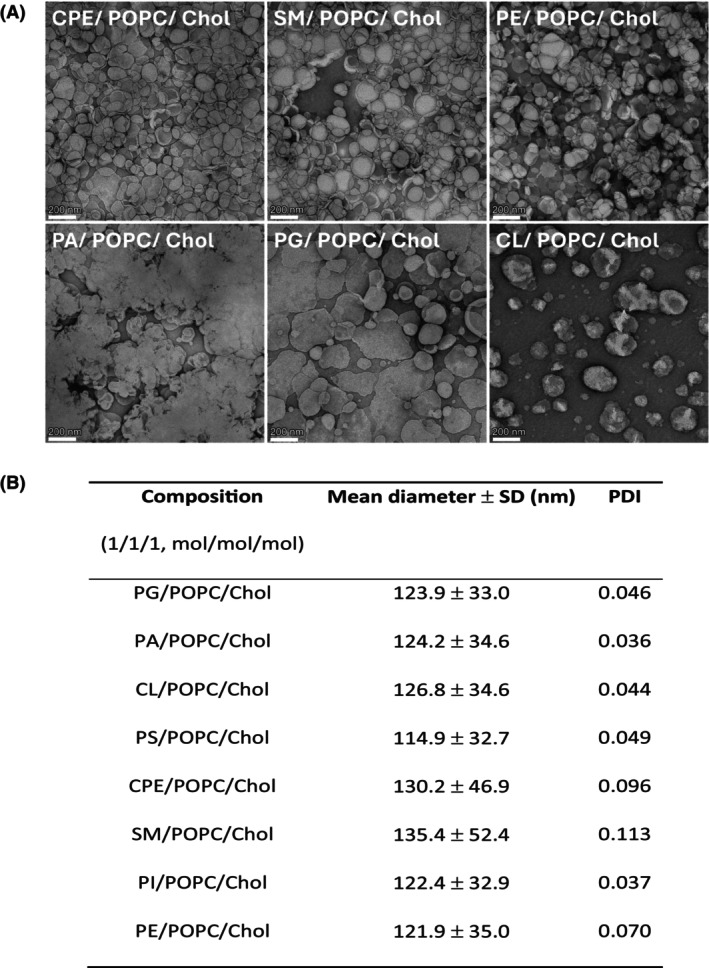
Determination of the size and shape of large unilamellar vesicles (LUVs) used in this study. (A) Determination of the LUVs' shape and size by transmission electron microscopy. (B) Determination of the mean diameter and polydispersity index (PDI) of LUVs by dynamic light scattering. The data represent the mean value of five independent measurements ± SD.

Although prone to various artifacts, transmission electron microscopy (TEM) is a convenient method that can provide basic information on the shape, size, and integrity of lipid vesicles [30]. The TEM images of selected negatively stained large unilamellar vesicles (LUVs) composed of equimolar ratios of 1‐palmitoyl‐2‐oleoyl‐*sn*‐glycero‐3‐phosphocholine (POPC), cholesterol and the third lipid component (Fig. 1A), shows that the addition of both sphingolipids (sphingomyelin or ceramide phosphoethanolamine (CPE)), as well as the neutral glycerophospholipid phosphatidylethanolamine, results in the formation of morphologically stable spherical vesicles whose diameters are comparable to the ones measured in the suspension (Fig. 1B). In contrast, the addition of negatively charged glycerophospholipids (phosphatidic acid or phosphatidylglycerol) results in the formation of apparently less stable vesicles that coalesce into larger structures. The formation of larger and fewer LUVs can also be observed when these are supplemented with CL, likely reflecting the preference of this glycerophospholipid for negatively curved membranes [9, 31, 32].

**Fig. 2:**
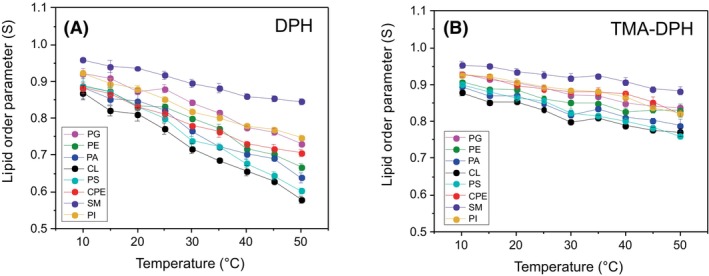
Lipid order parameter (S) calculated from the anisotropy of the fluorescent probes diphenylhexatriene (DPH) and trimethylammonium diphenylhexatriene (TMA‐DPH) of large unilamellar vesicles composed of equimolar ratios of POPC, cholesterol, and the third lipid component, as indicated on the graph. (A) Lipid order parameter calculated from the anisotropy of DPH. (B) Lipid order parameter calculated from the anisotropy of TMA‐DPH. The data represent the mean value of five independent measurements ± SD. The vesicles were suspended in 140 mm NaCl, 20 mm Tris, pH 7.4. Anisotropy was measured in the temperature range from 10 °C to 50 °C, with 5 °C intervals. CL, cardiolipin; CPE, ceramide phosphoethanolamine; PA, phosphatidic acid; PE, phosphatidylethanolamine; PG, phosphatidylglycerol; PI, phosphatidylinositol; POPC, 1‐palmitoyl‐2‐oleoyl‐*sn*‐glycero‐3‐phosphocholine; PS, phosphatidylserine; SM, sphingomyelin.

**Fig. 3:**
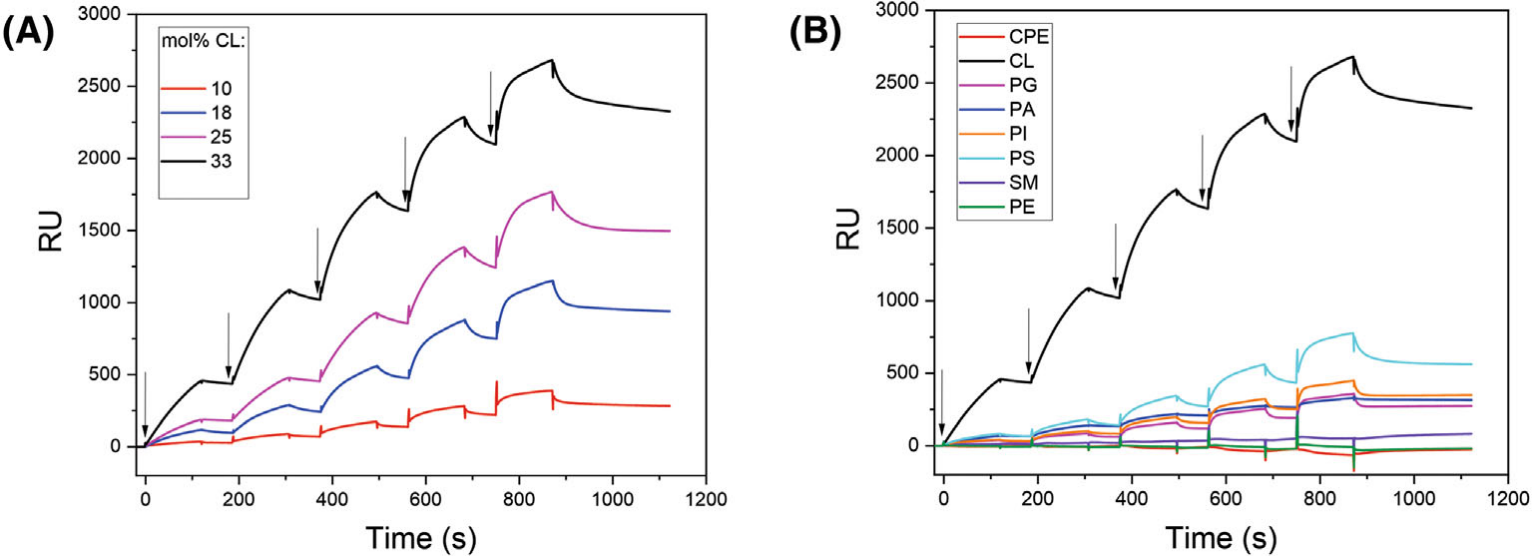
Binding of anti‐cardiolipin (CL) antibodies to immobilized large unilamellar vesicles (LUVs) with different lipid compositions. (A) Binding of anti‐CL antibodies to LUVs composed of equimolar ratios of POPC and cholesterol, and a varying CL molar content, as indicated. (B) Binding of anti‐CL antibodies to LUVs composed of equimolar ratios of POPC, cholesterol, and the third lipid component, as indicated, compared to binding to LUVs with 33 mol% CL (black curve, control).

In the colloid suspension, all the investigated LUV samples formed uniform vesicles, as indicated by PDI values below the threshold value of 0.2 at which the vesicle population is considered homogenous (Fig. 1B) [33]. Lipid order parameters (*S*) of the investigated LUVs in the temperature range from 10 °C to 50 °C, calculated from fluorescence anisotropy of two diphenylhexatriene (DPH)‐based lipid probes, are shown in Fig. 2. While DPH (Fig. 2A) reports the fluidity of the bilayer hydrophobic core, the fluorescence of its trimethylammonium derivative TMA‐DPH (Fig. 2B) is dependent on the fluidity of the outer regions of the bilayer [34]. The highest and comparable lipid order parameters at all tested temperatures were observed for sphingomyelin‐containing LUVs (0.917 and 0.926 with DPH and DPH‐TMA at 25 °C, respectively), which is consistent with this sphingolipid's tendency to tightly pack with cholesterol [35]. At both 25 °C and 37 °C, equimolar lipid mixtures composed of sphingomyelin, POPC, and cholesterol show phase separation and exist as mixtures of liquid‐ordered and liquid‐disordered states [36]. The lipid order parameter of LUVs containing a known EryA sphingolipid receptor, the CPE [37, 38], was lower and comparable to order parameters of LUVs supplemented with glycerophospholipids with two acyl chains. Indeed, in artificial bilayer membranes composed of CPE, phosphatidylcholine, and cholesterol, CPE does not induce the formation of liquid‐ordered domains, which are characteristic of sphingomyelin/phosphatidylcholine/cholesterol mixtures [39]. Membranes of LUVs supplemented with a tetra‐acylated glycerophospholipid CL showed the highest fluidity, assessed by both DPH and TMA‐DPH. In these LUVs, the value of the order parameter at 25 °C in membrane upper regions was 0.831, while the value around the acyl chains of the membrane dropped to 0.771. These results corroborate the previously reported ability of CL to decrease the membrane mechanical stability and increase its fluidity [32, 40, 41, 42]. Finally, the presence of CL at the membrane surface of our artificial lipid systems was confirmed by a strong binding of anti‐CL antibodies to these LUVs. This binding was directly proportional to the CL membrane content (Fig. 3A).

Although we could confirm that anti‐CL antibodies exhibit a preference for membrane CL, a certain degree of binding also was observed with LUVs supplemented with the same molar content of other negatively charged glycerophospholipids, in the following order: phosphatidylserine > phosphatidylinositol~phosphatidic acid~phosphatidylglycerol (Fig. 3B).

### Interaction of EryA‐mCherry and annexin V‐FITC with cardiolipin‐containing artificial lipid vesicles

#### Binding of EryA‐mCherry and annexin V‐FITC to large unilamellar lipid vesicles

**Fig. 4:**
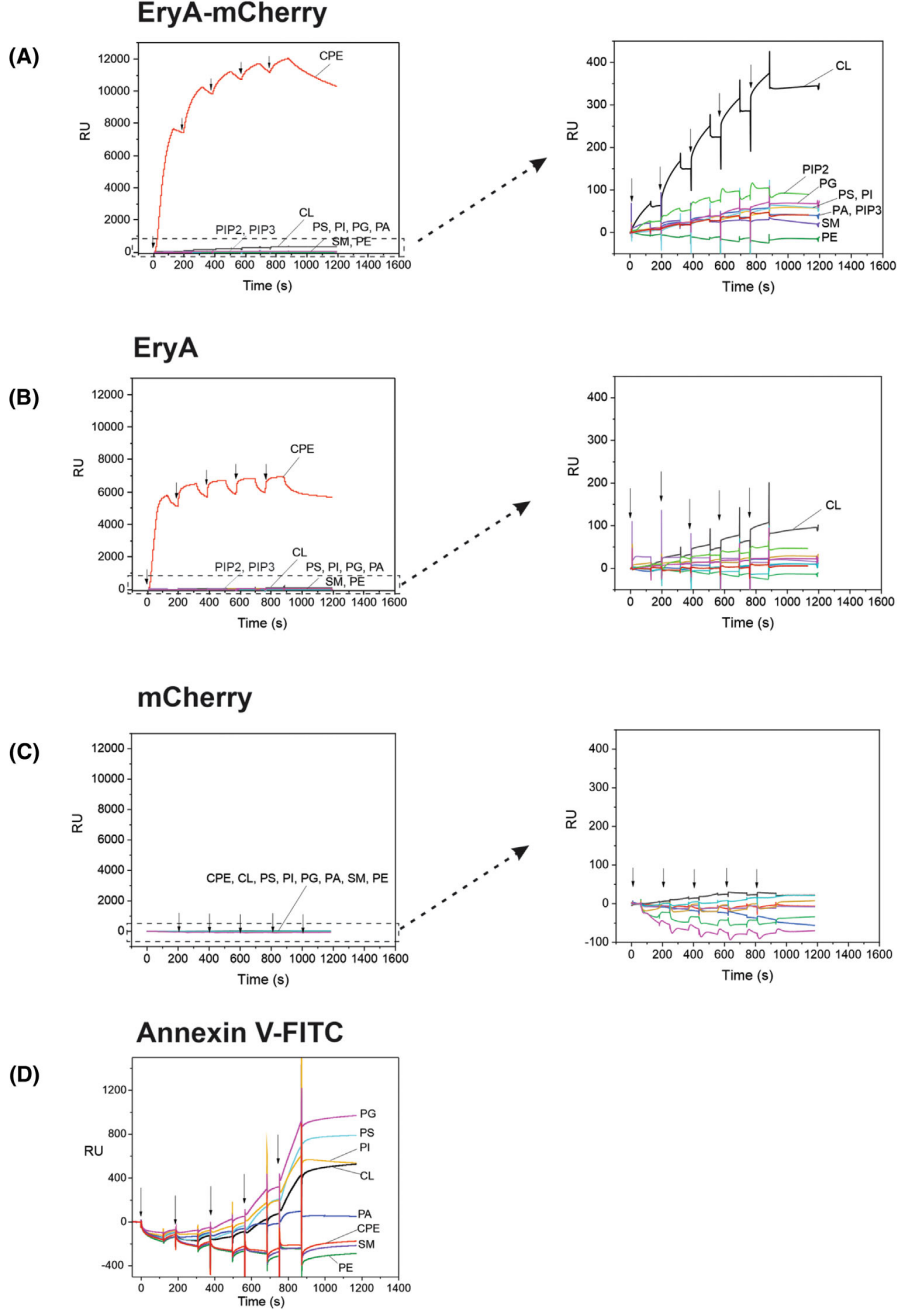
Binding of different proteins used in this study to immobilized equimolar large unilamellar vesicles composed of POPC, cholesterol, and the third lipid component, as indicated on the graph. The binding was assessed by surface plasmon resonance. (A) Binding of EryA‐mCherry. (B) Binding of EryA. (C) Binding of mCherry. (D) Binding of Annexin V‐FITC. Vesicles were immobilized on the Biacore L1 chip from 7500 to 11 500 response units (RU). Proteins were injected using the kinetic titration approach in a single cycle by successive injections (from left to right, as indicated by arrows) of 1, 2, 3, 4, and 5 μm (EryA, mCherry, or EryA‐mCherry), or 3.13, 6.25, 12.5, 25, and 50 nm (annexin V‐FITC) concentration. Right panels represent the magnification of the *Y*‐axis (0–400 RU) of the left panels. Representative sensorgrams from 10 and 2 independent experiments are shown for the binding of proteins to vesicles supplemented with CL, and for all other measurements, respectively. Spikes at the beginning and end of the injection result from an incomplete alignment of the sensorgrams when subtracting the reference flow cell from the active flow cell. CL, cardiolipin; CPE, ceramide phosphoethanolamine; PA, phosphatidic acid; PE, phosphatidylethanolamine; PG, phosphatidylglycerol; PI, phosphatidylinositol; PIP2, phosphatidylinositol (4,5)‐bisphosphate; PIP3, phosphatidylinositol (3,4,5)‐trisphosphate; POPC, 1‐palmitoyl‐2‐oleoyl‐sn‐glycero‐3‐phosphocholine; PS, phosphatidylserine; SM, sphingomyelin.

**Fig. 5:**
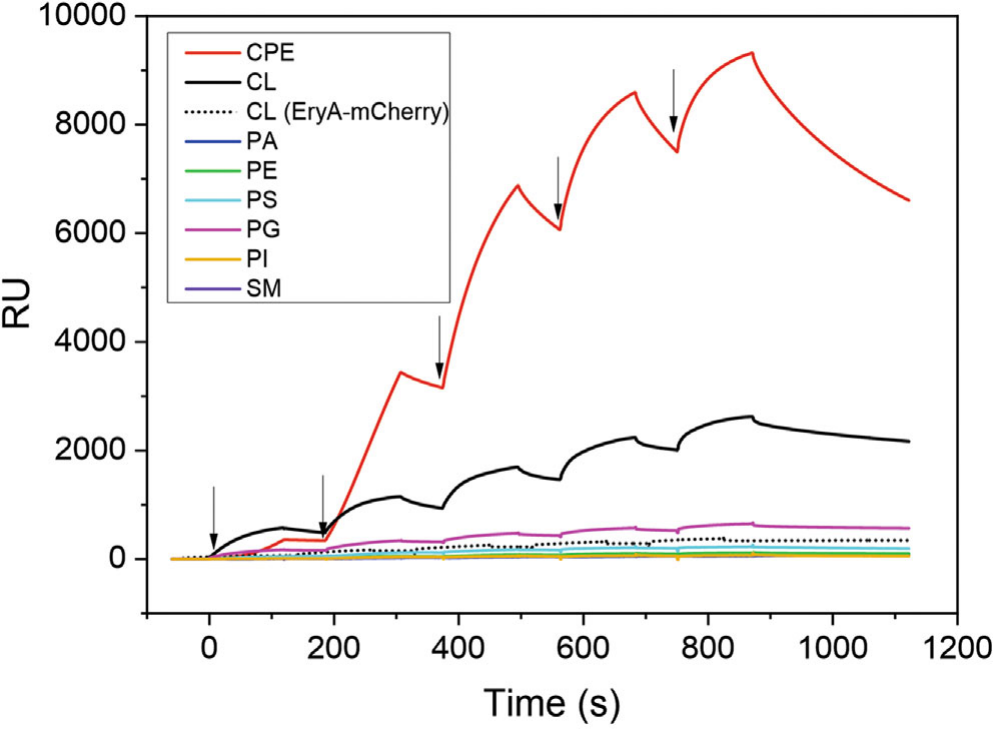
Binding of EryA‐EGFP to immobilized equimolar large unilamellar vesicles composed of POPC, cholesterol, and the third lipid component, as indicated on the graph. EryA‐EGFP was injected using the kinetic titration approach in a single cycle by successive injections (from left to right, as indicated by arrows) of 1, 2, 3, 4, and 5 μm protein concentration.

Surface plasmon resonance studies with LUVs immobilized on a chip revealed a selective and irreversible interaction of EryA‐mCherry with artificial lipid membranes composed of an equimolar mixture of POPC, cholesterol, and CL, which was not observed with LUVs supplemented with the same molar ratio of other mammalian membrane lipids, including those with two or three negative charges (Fig. 4A, right panel). Interestingly, the binding of EryA devoid of its fluorescence tag to CL‐containing LUVs was approximately 3.5‐fold lower (Fig. 4B, right panel). However, EryA is also smaller (14.96 kDa compared to 44.2 kDa for EryA‐mCherry) and therefore these response units are not directly comparable. Similar behavior was observed during the assessment of the binding of EryA‐mCherry or EryA to LUVs supplemented with CPE, an invertebrate‐specific high‐affinity membrane receptor for aegerolysins (Fig. 4A,B, left panels). mCherry by itself did not interact with any of the tested LUVs (Fig. 4C). Interestingly, when the same experiment was repeated with EryA in fusion with enhanced green fluorescent protein (EryA‐EGFP), the response was even 6.2‐fold higher than that of EryA‐mCherry (Fig. 5), while the sizes of the recombinant proteins are comparable (44.2 and 44.4 kDa for EryA‐mCherry and EryA‐EGFP, respectively). To further evaluate the binding of EryA‐mCherry to CL‐containing LUVs, we conducted multi‐cycle kinetic experiments, estimating a *K*_D_ value of 4.7 ± 1.6 μm. The binding of annexin V‐FITC, which is one of the most commonly used fluorescent probes for the determination of surface‐exposed phosphatidylserine during the early apoptosis, was considerably stronger than the binding of EryA‐mCherry (Fig. 4D), as the same degree of binding to CL‐containing LUVs was observed already at low nanomolar protein concentrations. However, the binding of annexin V‐FITC was found to be much less specific as this protein was able to bind to all LUVs supplemented with negatively charged phospholipids, in the following order of affinity: phosphatidylglycerol > phosphatidylserine > phosphatidylinositol > CL > phosphatidic acid. Annexin V‐FITC did not interact with LUVs supplemented with sphingolipids or neutral glycerophospholipids (Fig. 4D).

**Fig. 6:**
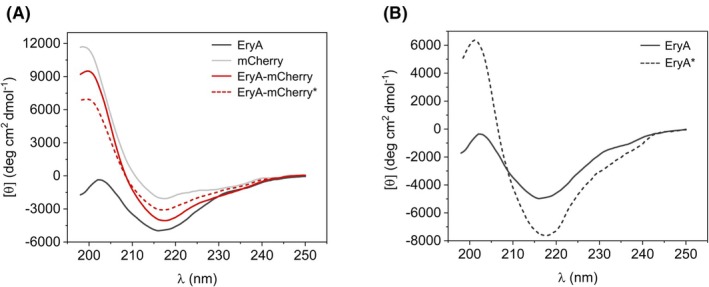
Circular dichroism spectra of EryA, mCherry, and EryA‐mCherry. (A) Measured spectra of the individual proteins EryA, mCherry, and EryA‐mCherry are shown with solid lines. Theoretical spectrum of the fusion protein (EryA‐mCherry*, dashed line), assuming no conformational changes in EryA and mCherry upon fusion, was calculated from the measured spectra of free proteins, as described in Materials and methods. (B) Comparison of the measured spectrum of free EryA (solid line) and the theoretical spectrum of the EryA part in the EryA‐mCherry fusion protein (EryA*, dashed line). The theoretical spectrum of EryA* was calculated from the measured spectra of EryA‐mCherry and free mCherry proteins, with the assumption that the mCherry conformation remains unchanged in the fusion protein. Representative spectra from three independent experiments are shown.

To gain better insight into the structural differences that might be involved in the observed enhanced membrane binding of EryA‐mCherry over EryA, the secondary structures of the investigated proteins (EryA, mCherry, EryA‐mCherry) were analyzed by CD‐spectropolarimetry (Fig. 6). The CD‐spectra of all the proteins revealed a characteristic β‐structure, in accordance with the published structures of mCherry (PDB id: 6YLM) and ostreolysin A6 (PDB id: 6MYI), an aegerolysin protein bearing a high (79%) amino acid identity with EryA. The comparison of the measured EryA‐mCherry fusion spectrum and the EryA‐mCherry spectrum that we calculated by combining the spectra of free EryA and free mCherry suggests that the conformation of one of the proteins (EryA or mCherry), or perhaps both, is altered in their fusion form. From these measurements, however, it is difficult to conclude which of the proteins changes its conformation in the fusion form. Assuming that the structure of mCherry remains unchanged, a rather significant increase in structural ordering of the EryA conformation in the fusion is visible. Further, the structure of EryA‐mCherry did not change when analyzed in complex with CL‐supplemented lipid vesicles (Fig. S1), indicating that the protein does not undergo significant conformational modifications upon interacting with these membranes.

#### Permeabilization of small unilamellar vesicles by EryA‐mCherry/PlyB and EryA/PlyB protein mixture

EryA‐mCherry was ineffective in permeabilizing the membranes of calcein‐loaded small unilamellar vesicles (SUVs) when supplemented with its pore‐forming protein partner pleurotolysin B (PlyB) (Fig. S2A). Weak permeabilization (15% and 12%) was observed only with the highest concentrations of EryA‐mCherry/PlyB complexes on SUVs containing CPE and phosphatidic acid, respectively. Interestingly, when EryA/PlyB complexes were added to these SUVs, a strong and selective permeabilization of CPE‐containing SUVs (IC_50_ = 30 nm), as already reported [38], was observed (Fig. S2B). EryA/PlyB complexes were unable to disrupt the membranes of any other lipid composition.

### Interaction of EryA‐mCherry with plasma membrane of healthy and apoptotic mammalian cells

In accordance with the obtained results of EryA‐mCherry interaction with artificial lipid membranes, the evaluation of its potential as a marker of apoptosis was tested on Madin‐Darby canine kidney epithelial (MDCK) cells and on human RT4 urothelial papilloma cells. Binding of EryA‐mCherry, annexin V‐FITC, and anti‐CL antibodies was assessed after cell treatment with an apoptosis inducer staurosporine (STS) or UV irradiation, and on untreated cells (control).

#### EryA‐mCherry labeling in apoptotic mammalian cells

**Fig. 7:**
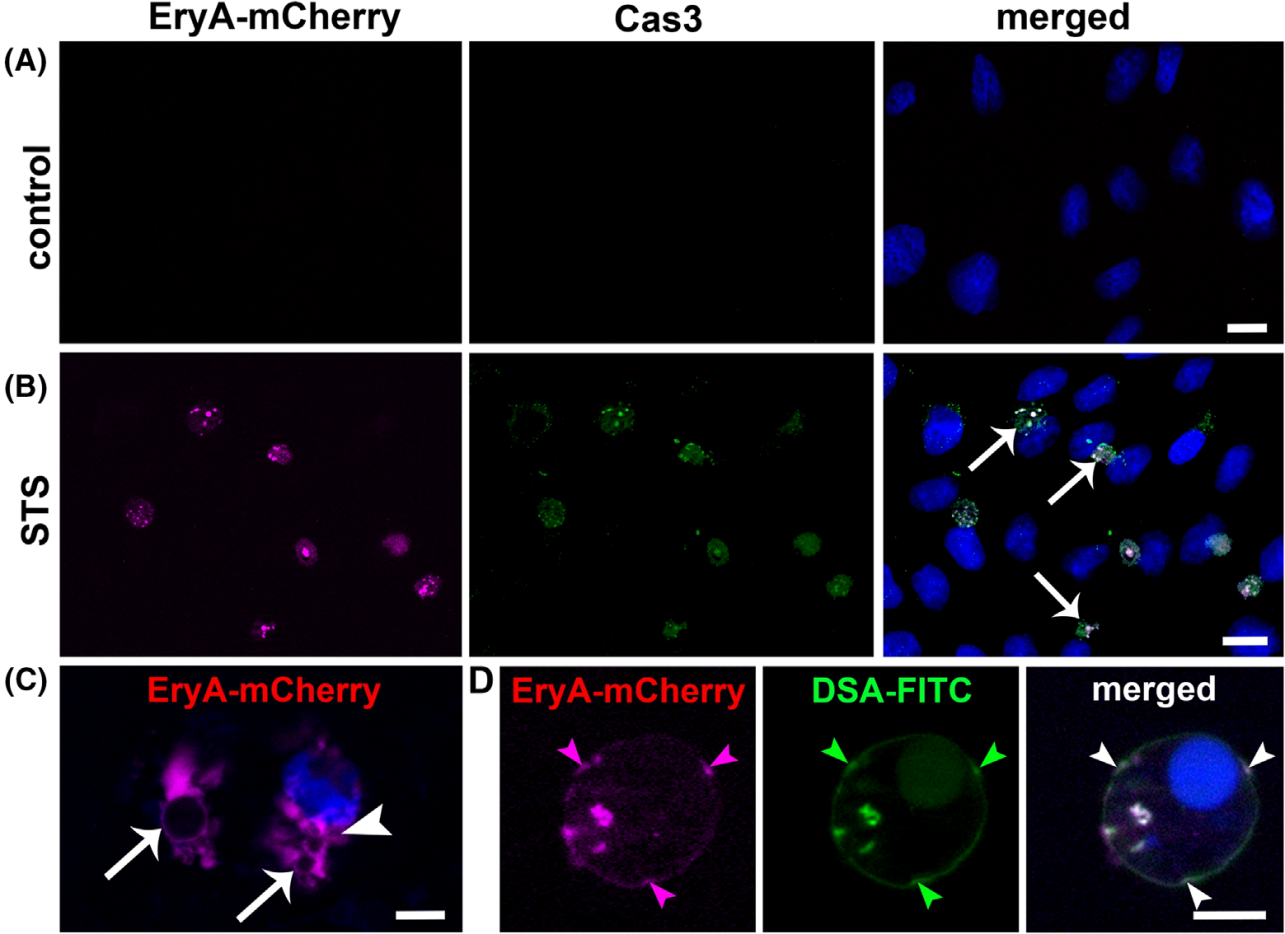
Representative fluorescence microscopy images of cell surface labeling of MDCK cells with EryA‐mCherry. Untreated MDCK cells (A) or MDCK cells treated with 2 μm staurosporine (STS) for 1 h (B) were successively labeled with 10 μm EryA‐mCherry (left panels) and with primary Cas3 antibodies (middle panels), as described in the Methods. Arrows indicate apoptotic cells, labeled with EryA‐mCherry and Cas3 antibodies (B, right panel). (C) Optical sectioning showing that EryA‐mCherry labels round structures, blebs (arrows) and cytoplasm (arrowhead) in apoptotic cells. (D) In the membrane of a bleb, EryA‐mCherry (magenta arrowheads) and DSA‐FITC (green arrowheads) colocalize (white arrowheads). Nuclei are stained with DAPI (blue). mCherry fluorescence is pseudocolored magenta to enhance accessibility for readers with red–green color vision deficiency. Scale bars: 20 μm (A, B) and 5 μm (C, D). Representative fluorescence images of MDCK cells labeled with EryA–mCherry, taken from at least three independent experiments, are shown. At least 200 cells per condition were analyzed across randomly selected fields (*n* = 200 cells).

In order to evaluate the potential of EryA‐mCherry as an apoptosis marker, we assessed its binding to the membranes of non‐treated and apoptotic MDCK cells (Fig. 7). Non‐apoptotic cells showed neither EryA‐mCherry (Fig. 7A, left panel), nor the apoptotic marker caspase 3 (Cas3) labeling (Fig. 7A, middle and right panel). Apoptosis was induced by treating the cells with 2 μm STS for 1 h and was confirmed with Cas3 (Fig. 7B, middle panel). Labeling showed that the Cas3‐positive cells were also positive for EryA‐mCherry labeling (Fig. 7B, left and right panel). In apoptotic cells, EryA‐mCherry labeling was uniform in the cytoplasm and on membranes of blebs (Fig. 7C, Fig. S3). The location of EryA‐mCherry on the plasma membrane was demonstrated by colocalization of EryA‐mCherry with FITC‐conjugated lectin DSA [43], which binds specific carbohydrate moieties on glycoproteins and glycolipids in the plasma membrane (Fig. 7D). Similar EryA‐mCherry labeling of the cytoplasm and membranes was observed in RT4 urothelial papilloma cells undergoing apoptosis after STS treatment (Fig. S4A,B) or after UV irradiation in RT4 cells (Fig. S4C) and MDCK cells (Fig. S4D).

#### Co‐labeling of EryA‐mCherry and anti‐CL antibodies in MDCK cells undergoing apoptosis

**Fig. 8:**
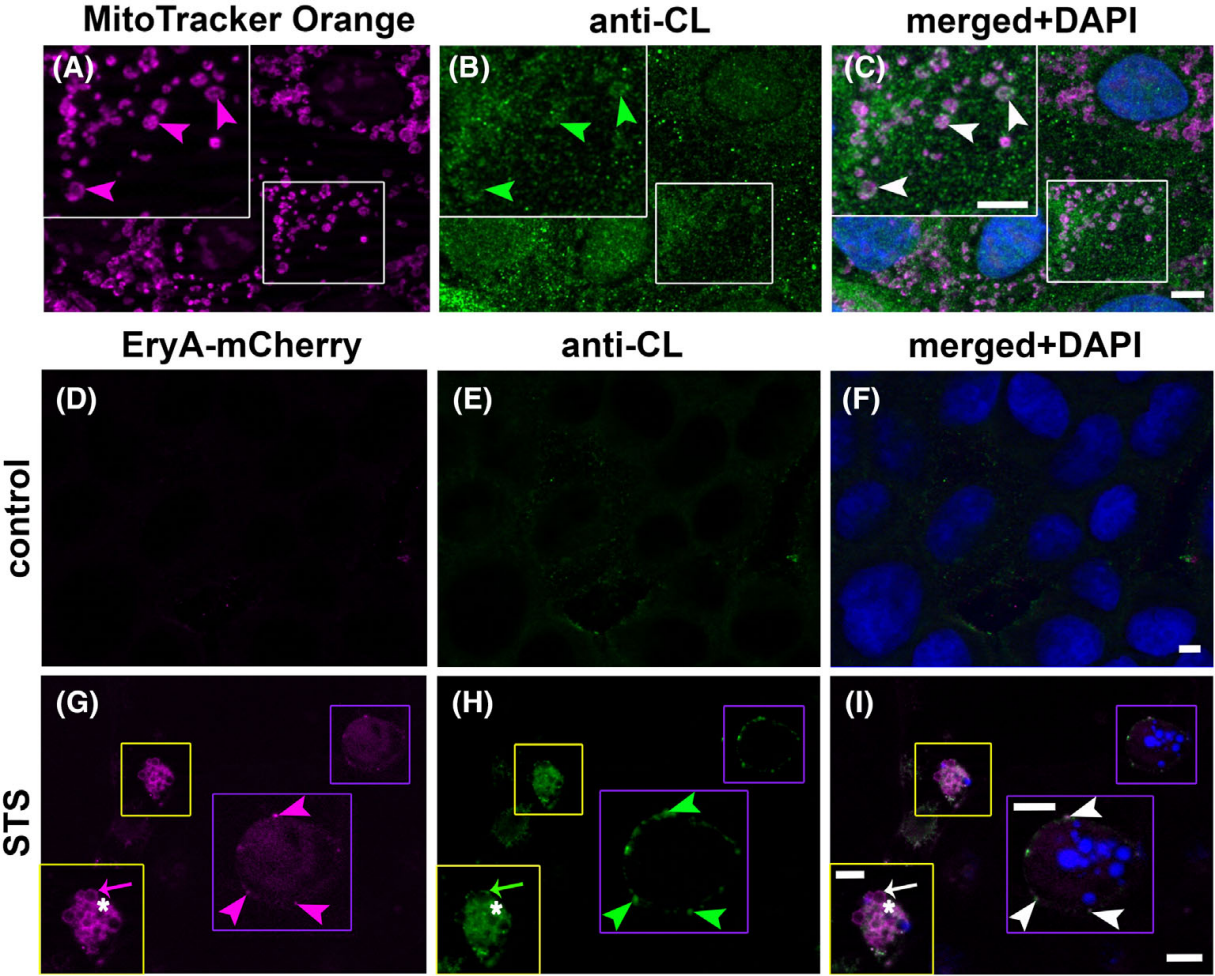
Representative fluorescence microscopy images of cell surface labeling of apoptotic MDCK cells with EryA‐mCherry and anti‐cardiolipin (CL) antibodies. In untreated MDCK cells, mitochondria indicated by MitoTracker labeling (A, magenta arrowheads) and CL, indicated by anti‐CL immunolabeling (green arrowheads, B) were colocalized (C, white arrowheads). Untreated MDCK cells (D–F) or MDCK cells treated with 2 μm staurosporine (STS) for 1 h (G–I) were successively labeled with 10 μm EryA‐mCherry (left panels) and with primary anti‐CL antibodies (middle panels), as described in the Methods. (G) EryA‐mCherry labels the membranes of blebs (yellow frame, magenta arrow), cytoplasm (yellow frame, asterisk), and the membrane of apoptotic cells (magenta frame, magenta arrowheads). (H) Anti‐CL antibody labeling is present in membranes of blebs (yellow frame, green arrow), cytoplasm (yellow frame, asterisk) and in the membrane of apoptotic cells (magenta frame, green arrowheads). (I) EryA‐mCherry and anti‐CL antibody co‐label the membranes of blebs (yellow frame in the left corner, white arrow), cytoplasm (yellow frame in the left corner, asterisk) and the membrane of apoptotic cells (magenta frame, white arrowheads). Apoptotic membranes show patchy EryA‐mCherry and anti‐CL co‐labeling (I, white arrowheads). Note that apoptotic cells are in a different focus plane than the underlying non‐apoptotic cells. Larger colored frames are higher magnifications of corresponding smaller colored frames. Nuclei are stained with DAPI (blue). mCherry fluorescence is pseudocolored magenta to enhance accessibility for readers with red–green color vision deficiency. Scale bars: 5 μm. Representative fluorescence images of MDCK cells labeled with EryA–mCherry and anti‐CL antibodies, taken from at least three independent experiments, are shown. At least 200 cells per condition were analyzed across randomly selected fields (*n* = 200 cells).

In the control MDCK cells, anti‐CL antibodies successfully recognized mitochondrial CL, as revealed by MitoTracker and anti‐CL antibodies double labeling (Fig. 8A–C). In contrast to the absence of binding of both EryA‐mCherry and anti‐CL antibodies to cell membranes of the control MDCK cells (Fig. 8D–F), successive EryA‐mCherry labeling (Fig. 8G) and anti‐CL immunolabeling (Fig. 8H) showed the ability of both proteins to bind to apoptotic MDCK cells and their budding plasma membrane (Fig. 8I). EryA‐mCherry could be observed on the membranes of blebs and in the cytoplasm of apoptotic cells (Fig. 8G). In these apoptotic cells, EryA‐mCherry (Fig. 8G) and anti‐CL antibodies (Fig. 8H) co‐labeling was present at the same membrane regions (Fig. 8I).

#### Co‐labeling of EryA‐mCherry and annexin V‐ FITC in apoptotic MDCK cells

**Fig. 9:**
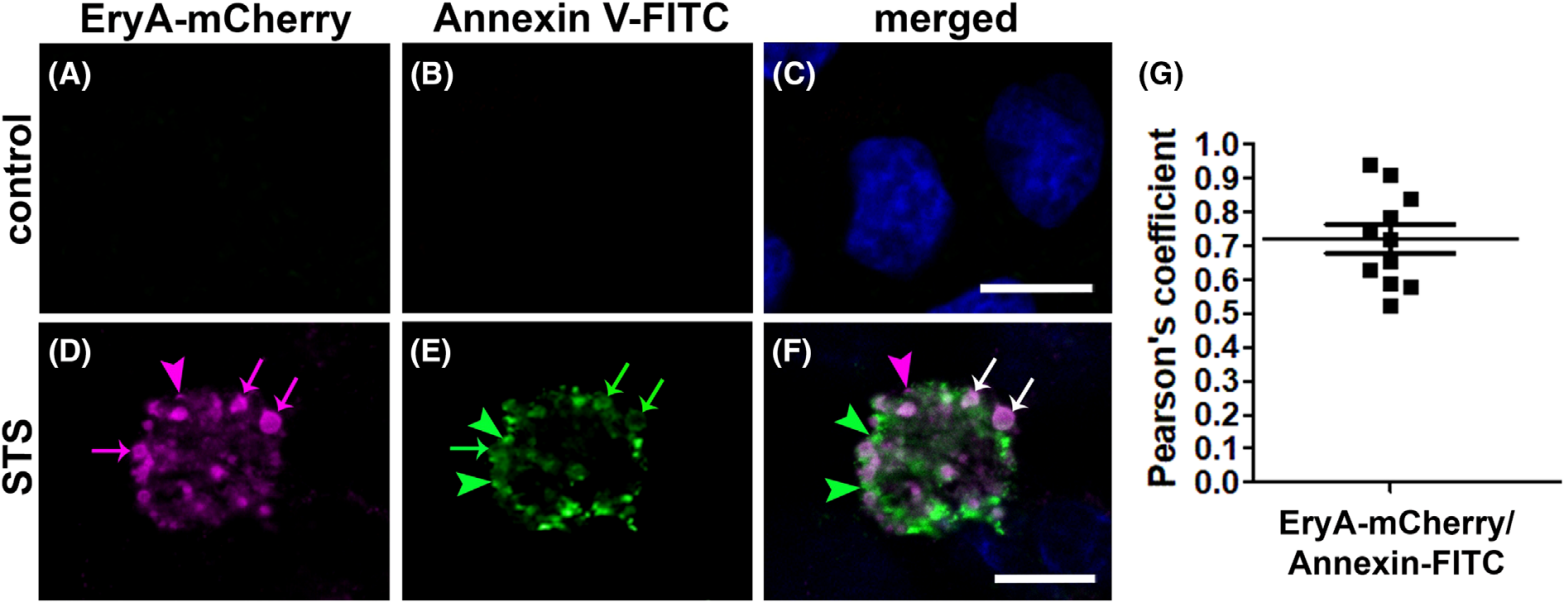
Representative fluorescence microscopy images of cell surface labeling of apoptotic MDCK cells with EryA‐mCherry and annexin V‐FITC. Untreated MDCK cells (A–C) or MDCK cells treated with 2 μm staurosporine (STS) for 1 h (D–F) were double labeled with 10 μm EryA‐mCherry (left panels) and annexin V‐FITC (middle panels), as described in the Methods. EryA‐mCherry (D, magenta arrows) and annexin V‐FITC (E, green arrows) bind to the membranes of apoptotic cell blebs. EryA‐mCherry labels membrane regions (D, F, magenta arrowheads) which are distinctive from annexin V‐FITC labeling (E, F, green arrowheads). EryA‐mCherry and annexin V‐FITC also co‐label membranes of blebs (F, white arrows). Nuclei are stained with DAPI (blue). mCherry fluorescence is pseudocolored magenta to enhance accessibility for readers with red–green color vision deficiency. Scale bars: 10 μm. Representative fluorescence images of MDCK cells labeled with EryA–mCherry and Annexin–FITC, taken from at least three independent experiments, are shown. At least 200 cells per condition were analyzed across randomly selected fields (*n* = 200 cells). (G) The quantification of EryA‐mCherry and annexin V‐FITC colocalization, presented as Pearson's correlation coefficient, measured on optical sections. Shown is average value ± SEM.

Similarly to the binding of EryA‐mCherry and anti‐CL antibodies (Fig. 8), EryA‐mCherry and annexin V‐FITC did not bind to cell membranes of the control MDCK cells (Fig. 9A–C). In contrast, EryA‐mCherry (Fig. 9D) and annexin V‐FITC (Fig. 9E) presumably labeled the same membrane regions of blebs (Fig. 9F) in apoptotic MDCK cells. Membrane regions with distinctive EryA‐mCherry and with distinctive annexin V‐FITC labeling were also revealed (Fig. 9D–F). Quantitative analysis of the colocalization between EryA‐mCherry and annexin V‐FITC showed that Pearson's correlation coefficient was 0.721 (±0.042), which is categorized as strong (0.49–0.84) colocalization level [44] (Fig. 9G).

## Discussion

Proteins from the aegerolysin family, in particular those produced by the edible oyster mushrooms (genus *Pleurotus*), have been drawing the attention of researchers due to their specific interaction with membrane lipids and lipid domains [37, 45, 46]. The common high‐affinity receptor of all these aegerolysins is the invertebrate‐specific membrane sphingolipid, CPE [37, 38]. This feature, combined with the ability of membrane‐bound aegerolysins to form bicomponent pore‐forming complexes with their larger protein partner PlyB bearing a membrane‐attack complex/perforin (MACPF) domain, has been exploited to develop new environmentally friendly and highly specific bioinsecticides [38, 47]. Additionally, selected aegerolysins can sense mammalian‐specific sphingolipid sphingomyelin in complex with cholesterol [38, 48, 49], and have been proposed as markers of lipid rafts [50], while aegerolysin/PlyB complexes can be selectively toxic to cancer cells that express elevated levels of lipid rafts in their membranes [51].

### EryA‐mCherry specifically binds to artificial lipid membranes containing CL

To date, EryA is the only described *Pleurotus* aegerolysin binding exclusively to the invertebrate‐specific sphingolipid CPE, and not to sphingomyelin [37, 38]. In 2021, a fluorescently fused recombinant EryA‐EGFP has been shown to bind another lipid target in membranes of artificial vesicles and in bacterial cells, the CL, at physiological pH values [29]. This affinity for CL has also been reported for ostreolysin A6 from *P. ostreatus*, and for aegerolysins from some other basidiomycetes, but exclusively at lower pH values, and this binding occurred at approximately 1000‐fold higher concentrations than the binding of EryA‐EGFP [52].

Our experiments with artificial cell membranes supplemented with different mammalian membrane lipids confirmed the findings of Sakihara *et al*. [29], and showed the ability of EryA, fused with mCherry, to selectively interact with CL‐containing membranes at physiological pH values. Interestingly, this binding was reduced when the protein was tested without its fluorescent tag, mCherry, and was absent when only mCherry was applied. It remains unclear whether this difference reflects the smaller size of the untagged protein resulting in lower response units in the assay, or is due to the genuinely lower binding of EryA alone. In the latter case, the observed difference could be attributed to a conformational effect, since our CD experiments suggest that the fusion protein could adopt the conformation that is better suited for interaction with membrane CL. Further, EryA‐mCherry could not bind lipid membranes supplemented with phosphoinositides bearing two or three additional negative charges, so its binding seems to be independent on membrane lipid surface charge. Finally, although occurring at micromolar concentrations, this EryA‐mCherry binding to CL is much more specific than the binding of annexin V‐FITC, which binds the same membranes in low nanomolar concentrations but shows a broader specificity for all negatively charged membrane glycerophospholipids ([53]; this work).

Furthermore, when tested in concert with its MACPF‐domain partnering protein PlyB, neither EryA‐mCherry nor EryA could permeabilize lipid vesicles containing CL. The same behavior was already observed for other aegerolysin/PlyB pairs from higher fungi [52], pointing to the importance of a sphingolipid membrane receptor for the successful formation of the pore. The underlying reasons for this lack of permeabilization can be attributed to the interactions of different protein amino acid residues with sphingolipids compared to CL, or to different membrane lipid packing of membranes containing CL, which in turn hinders the EryA(‐mCherry)/PlyB interaction and the consecutive pore formation. Indeed, our experiments confirm the high degree of fluidity of CL‐containing lipid systems in contrast to membranes supplemented with other mammalian lipids, as also reported in previous studies [40, 41, 42]. Interestingly, specific and effective permeabilization of lipid vesicles containing the EryA high‐affinity lipid receptor CPE was observed only with the EryA/PlyB protein mixture, and not with EryA‐mCherry/PlyB. These results suggest that the mCherry tag hinders the membrane permeabilization activity of EryA‐mCherry/PlyB complexes, likely by preventing the interaction of the pore‐forming component PlyB with its lipid‐bound aegerolysin partner.

### EryA‐mCherry specifically labels CL in apoptotic mammalian epithelial cells

In eukaryotic cells during apoptosis, CL is redistributed from its primary location in mitochondria to the plasma membrane, thus playing an important role as a marker of apoptosis [13, 14, 15]. In our experiments with MDCK cells, EryA‐mCherry exclusively labeled cells undergoing apoptosis, as revealed by double labeling with anti‐Cas3 antibodies as markers of apoptosis, and EryA‐mCherry. Our microscopy analyses clearly demonstrated that EryA‐mCherry was present in the cytoplasm and on the membranes of apoptotic cells, which were labeled with a FITC‐conjugated lectin DSA, known for its strong affinity for specific carbohydrate moieties on plasma membrane glycoproteins and glycolipids [43, 54]. EryA‐mCherry showed similar labeling of the cytoplasm and membranes of apoptotic blebs in both MDCK and RT4 cells, derived from kidney and urinary bladder epithelia, respectively. Further, this labeling was similar regardless of whether apoptosis was induced chemically (by STS) or physically (by UV irradiation). Notably, EryA‐mCherry labeled the same membrane regions of blebs as anti‐CL antibodies, confirming EryA‐mCherry specificity for CL‐enriched membrane domains. Similar enrichment of CL domains, detected by the use of anti‐CL antibodies, was found in membranes of blebs in the pro‐monocytic cell line [14].

Annexins are calcium‐dependent anticoagulant proteins most commonly utilized in apoptosis assays due to their specific binding to negatively charged phosphatidylserine species, which are externalized on the plasma membrane during the process of apoptosis [53, 55]. However, the specificity of annexins is not restricted to phosphatidylserine species, as these proteins were shown to interact with several other negatively charged glycerophospholipids to a similar extent ([53]; this work). We were able to show that EryA‐mCherry is co‐localized with annexin V‐FITC at the plasma membrane of apoptotic cells. EryA‐mCherry and annexin V‐FITC were detected in distinct membrane domains, as well as in the same domains of apoptotic blebs. According to Pearson's coefficient measurement, this colocalization of EryA‐mCherry and annexin‐FITC is considered a strong one [44]. These results, also supported by surface plasmon resonance measurements on artificial lipid membranes, confirm the broader lipid affinity of annexin V‐FITC over EryA‐mCherry that recognizes exclusively CL. Although CL externalization was reported to precede that of the phosphatidylserine [13, 14, 15], we could not detect the EryA‐mCherry signal before annexin V‐FITC in apoptotic MDCK cells. This might be due to the low CL concentration in the plasma membrane during the early apoptosis events.

## Conclusions

Apoptosis plays a crucial role in various fields of biological research, yet there is a notable shortage of techniques that allow for continuous, non‐invasive, and nontoxic monitoring of this process. Sensitive and selective markers of apoptosis are still desirable, despite the many methods available. Cell labeling with the fluorescently fused nontoxic aegerolysin protein EryA offers a reliable, fast, and straightforward approach for studying and quantifying CL on the surface of apoptotic cells without their prior fixation. The data presented here indicate that the EryA‐mCherry and EryA‐EGFP are potential markers for the detection of apoptotic cells and markers for CL in other biological membranes and artificial membrane systems.

## Materials and methods

### Materials

#### Chemicals

All chemicals used in the present study were from Sigma–Aldrich (St. Louis, MO, USA) unless specified otherwise. Benzoase, RNAse, and protease inhibitors were from Merck, Darmstadt, Germany. Lipids used for preparation of artificial membrane systems (porcine brain sphingomyelin (SM), bovine liver l‐α‐phosphatidylinositol (PI), phosphatidylinositol (4,5)‐bisphosphate (PIP2), phosphatidylinositol (3,4,5)‐trisphosphate (PIP3), 1‐palmitoyl‐2‐oleoyl‐*sn*‐glycero‐3‐phosphocholine (POPC), chicken egg l‐α‐phosphatidylethanolamine (PE), bovine heart cardiolipin (CL), 2‐dioleoyl‐*sn*‐glycero‐3‐phosphate (phosphatidic acid; PA), 1‐palmitoyl‐2‐oleoyl‐*sn*‐glycero‐3‐phospho‐l‐serine (PS), chicken egg l‐α‐phosphatidylglycerol (PG), ovine cholesterol (Chol), and porcine brain ceramide phosphoethanolamine (CPE)) were from Avanti Polar Lipids (Alabaster, AL, USA). These lipids were stored at −80 °C and dissolved in chloroform prior to use. Ceramide phosphoethanolamine was dissolved in 1 mL chloroform/methanol (9/1, v/v).

#### Cells

Madin‐Darby canine kidney epithelial cells (MDCK: RRID CVCL_0422) and human urothelial papilloma cells (RT4: RRID CVCL 0036), both purchased from ATCC, were routinely cultured in an advanced Dulbecco's modified Eagle's/F12 growth medium (1/1, v/v) with 5% fetal bovine serum, 100 U·mL^−1^ penicillin, and 10 000 μg·mL^−1^ streptomycin. Cells were maintained in a humidified atmosphere at 37 °C and 5% CO_2_ and subcultured when at 90% confluence. Apoptosis was induced in MDCK and RT4 cells at 70% confluence by treating the cells with 2 μm STS (Cell Signaling, Danvers, MA, USA #9953) for 1 h at 37 °C or by exposing the cells to ultraviolet (UV) irradiation for 1 min in a safety cabinet. MDCK and RT4 cells were free of *Mycoplasma*, which was tested using the MycoAlert mycoplasma detection kit (Lonza, Basel, Switzerland). The culture media and supplements were purchased from Gibco, Invitrogen (Vienna, Austria).

#### Proteins and dyes

Primary human anti‐CL antibodies were from US Biological (Salem, MA, USA), and secondary goat anti‐human AlexaFluor‐488 antibodies were from Thermo Fisher Scientific (Waltham, MA, USA). Annexin V‐FITC was purchased from Merck Millipore (Darmstadt, Germany), and Cas3 antibodies were from Abcam (Abcam, Cambridge, UK). *Datura stramonium* lectin (DSA)‐FITC was from Vector Laboratories (Burlingame, CA, USA), and MitoTracker Orange CMTMRos (M7510) was from Thermo Fisher Scientific. The recombinant proteins EryA, EryA‐mCherry, EryA‐EGFP, mCherry, and PlyB were expressed in the *Escherichia coli* BL21(DE3) strain as described previously [37, 38, 48, 56]. They were further purified using fast liquid protein chromatography ÄKTA pure 25 (GE Healthcare, Uppsala, Sweden) and HiLoad™ 25/600 Superdex 75 pg (size exclusion) column (GE Healthcare), analyzed with sodium dodecyl sulphate polyacrylamide gel electrophoresis (SDS/PAGE; Bio‐Rad, Hercules, CA, USA), and stained with SimplyBlue SafeStain (Thermo Fisher Scientific). The proteins were concentrated using Amicon ultra‐15 (Ultracel‐10k, Millipore, Merck, USA) centrifugal filters, and their concentrations were determined using the Pierce™ BCA Protein Assay Kit.

### Methods

#### Preparation of artificial lipid vesicles

Lipids in different molar ratios (final concentration, 5 mg·mL^−1^) were used to prepare multilamellar vesicles in 140 mm NaCl, 20 mm Tris, pH 7.4, as described in [57], with the addition of 1 mm EDTA for the vesicle permeabilization assay and 2.5 μm CaCl_2_ for the surface plasmon resonance‐based binding studies. The suspensions of these multilamellar vesicles were subjected to five freeze–thaw cycles, diluted to the concentration of 1 mg·mL^−1^, and then extruded through 100 nm polycarbonate filters (Millipore, Burlington, MA, USA) at ~ 40–60 °C. The morphology of the obtained LUVs was analyzed by TEM and by dynamic light scattering on a Zetasizer Nano ZS (Malvern Instruments Ltd., Malvern, UK), as reported [57]. For the vesicle permeabilization assay, SUVs loaded with calcein at the self‐quenching concentration (80 mm) were prepared by sonication (20 min at an amplitude of 40%, with an interval of 10 s pulse and 10 s pause) of multilamellar vesicles.

#### Transmission electron microscopy

Four μL of the LUV suspension (1 mg·mL^−1^) in 140 mm NaCl, 20 mm Tris, pH 7.4 was left for 3 min on a formvar and carbon‐coated copper grid prepared with glow discharge. Afterwards, the excess liquid was blotted with filter paper, stained with 1% (w/v) uranyl acetate, its excess blotted with filter paper followed by drying of the grids in air. Lipid structures were visualized using the transmission electron microscope TALOS L120C (ThermoFisher Scientific, The Netherlands) operating at 100 kV. The data were collected with a CCD camera Ceta 16M using velox software.

#### Fluorescence emission anisotropy measurements

Fluorescence emission anisotropy (*r*) of DPH and TMA‐DPH probes was measured in samples of LUVs with different lipid compositions in 10‐mm path length cuvettes using Cary Eclipse fluorescence spectrophotometer (Agilent, Santa Clara, CA, USA). The measurements were taken over temperature range from 10 °C to 50 °C in 5 °C intervals with equilibration time of 5 min for each step, using Varian auto polarizers with slit widths with a nominal band‐pass of 5 nm for both excitation and emission. 2.5 μL of 1 mm DPH or 2 mm TMA‐DPH solution dissolved in dimethyl sulfoxide were added to 2.5 mL of LUVs (0.1 mg·mL^−1^) in 140 mm NaCl, 20 mm Tris, pH 7.4, with final DPH or TMA‐DPH concentration of 1 or 2 μm, respectively. Measurements of fluorescent anisotropy were done at excitation wavelength of 358 nm and the emission wavelength of 410 nm for both DPH and TMA‐DPH probes. The G‐factor (ratio of sensitivities of the detection system for vertically and horizontally polarized light) was always between 1 and 1.05 and was measured for each individual sample. The anisotropy was calculated with instrument's software using the following Eqn (1):

(1) $$r=\frac{I_{∥}-I_{⊥}}{I_{∥}+2I_{⊥}}$$

where $I_{∥}$ and $I_{⊥}$ are the parallel and perpendicular emission intensities, respectively. Lipid order parameter (*S*) was calculated using the following analytical expression (2) [58]:

(2) $$S=\frac{{\left[1-2\left(r/r_{0}\right)+5{\left(r/r_{0}\right)}^{2}\right]}^{1/2}-1+r/r_{0}}{2\left(r/r_{0}\right)}$$

where $r_{0}$ is the fluorescence anisotropy value of DPH in the absence of any rotational motion of the probe. The theoretical value of $r_{0}$ of DPH is 0.4, while the experimental values of $r_{0}$ are between 0.362 and 0.394 [59]. For our calculations, we used experimental value of *r*_0_ = 0.370 for DPH. The fluidity may be defined as the reciprocal of the lipid order parameter *S* [60].

#### Circular dichroism (CD) spectropolarimetry

The circular dichroism (CD) spectra were recorded with a CD spectrometer (J‐1500; JASCO, Tokyo, Japan). Spectra were scanned from 250 to 198 nm at 25 °C, with scanning speed set at 20 nm·min^−1^ and bandwidth at 1.0 nm. The CD signal of each sample was scanned three times, averaged, and smoothed with a Savitzky–Golay filter. A quartz cuvette with 1 mm optical path was used for all measurements. Protein concentrations were 0.2 mg·mL^−1^ (EryA and EryA‐mCherry) or 0.15 mg·mL^−1^ (mCherry). In the samples with LUVs containing 33 mol% CL, the total lipid concentration was 2.0 mg·mL^−1^. All samples were in 20 mm Tris/HCl (pH 7.4), 140 mm NaCl buffer. The CD signal of the buffer was subtracted from the signals of samples containing the proteins without LUVs. The CD signal of LUVs in buffer was subtracted from the signals of samples containing the proteins and LUVs. Mean residue weights of 109.19, 113.15, and 111.64 were used to calculate the molar ellipticities of EryA, mCherry and EryAmCherry, respectively. The theoretical CD‐spectrum of EryA‐mCherry fusion was calculated as a weighted sum of the molar ellipticities of free EryA and free mCherry, using weights of 0.36 for EryA (14.96 kDa) and 0.64 for mCherry (26.59 kDa). The theoretical spectrum of EryA in EryA‐mCherry fusion was calculated by subtracting the molar ellipticity of free mCherry from the experimentally determined molar ellipticity of EryA‐mCherry fusion, using the corresponding weights for EryA and mCherry as described above.

#### Surface plasmon resonance‐based binding studies

Interactions of the tested proteins (EryA‐mCherry, EryA, mCherry, EryA‐EGFP, annexin V‐FITC, anti‐CL antibodies) with LUVs with different lipid compositions were monitored using a surface plasmon resonance‐based refractometer (Biacore T‐200; GE Healthcare, Chicago, IL, USA) and a L1 sensor chip, using 20 mm Tris, 140 mm NaCl, 2.5 μm CaCl_2_, pH 7.4 as running buffer. The chip was cleaned with regeneration solutions (sodium dodecyl sulphate, octyl‐β‐d‐glucopyranoside, 30% ethanol with 1‐min injections at a flow rate of 30 μL·min^−1^), prior to the attachment of LUVs (1 mg·mL^−1^; 400‐s injections at a flow rate of 3 μL·min^−1^) with different lipid compositions to the second flow cell of the sensor chip to achieve responses of from 7500 to 11 500 RU. Equimolar POPC/cholesterol LUVs were injected across the first flow cell to control for possible non‐specific binding of the proteins to the chip dextran matrix.

To further minimize the non‐specific binding of the investigated proteins to the chip surface, an injection of 0.1 mg·mL^−1^ bovine serum albumin for 1 min at a flow rate of 30 μL·min^−1^ was performed prior to the addition of these proteins. Then, single‐cycle kinetic experiments were performed to test the interactions of EryA‐mCherry, EryA‐EGFP, EryA, or mCherry (1, 2, 3, 4, and 5 μm) or annexin V‐FITC (3.13, 6.25, 12.5, 25, and 50 nm) with 2‐min injections at a flow rate of 10 μL·min^−1^. The same was done for the binding of anti‐CL antibodies (25×, 50×, 100×, 200×, and 400× dilution) to immobilized large unilamellar vesicles composed of CL, POPC, and cholesterol with different CL molar content (10%, 18%, 25%, or 33%). To determine the K_D_ value of the EryA‐mCherry interaction with CL, multi‐cycle kinetic experiments were performed. In this case, LUVs were further diluted (approximately to 0.1 mg·mL^−1^) to achieve responses from 2500 to 3000 RU and injected for 50 s at a flow rate of 3 μL·min^−1^. Thereafter, to further reduce non‐specific binding resulting from the higher dilution and shorter injection times of LUVs, the sensor chip surfaces were additionally blocked by coating all cells with equimolar POPC/Chol LUVs. EryA‐mCherry (1.25, 2.5, 3.75, 5, 7.5, 10, 15, 20, 30, 40, 60, and 80 μm) was injected for 600 s at a flow rate of 20 μL·min^−1^ and the dissociation time was also increased to 600 s. Chip regeneration was performed using 1‐min injections of 0.5% sodium dodecyl sulfate, 40 mm β‐d‐glucopyranoside, 30% ethanol, and 100 mm NaOH at a flow rate of 10 μL·min^−1^. Experiments were performed in triplicates at 25 °C. Data were processed using biaevaluation software (GE Healthcare, Chicago, IL, USA). The *K*_D_ value was estimated by steady state affinity analysis.

#### Permeabilization of the small unilamellar vesicles

Permeabilization of calcein‐loaded SUVs after the addition of EryA/PlyB or EryA‐mCherry/PlyB complexes at a 12.5/1 molar ratio (EryA and EryA‐mCherry concentrations range from 0 to 5 μm) was determined as described [57] using a fluorescence microplate reader (Infinite NANO, Tecan, Männedorf, Switzerland) with excitation and emission set at 485 and 535 nm, respectively. The experiments were run for 20 min at 25 °C and were performed in triplicates. The permeabilization induced by lytic aegerolysin complexes was expressed as a percentage of maximal permeabilization obtained after the addition of Triton‐X 100 at a final concentration of 1 mm.

#### Labeling of MDCK and RT4 cells

MDCK cells (seeded at 3 × 10^4^ cells·cm^−2^) and RT4 cells (seeded at 5 × 10^4^ cells·cm^−2^) were grown for 2 days on glass coverslips and then treated with 2 μm STS for 1 h at 37 °C or with UV irradiation for 1 min at room temperature. For the control, we used untreated cells, followed by the labeling procedure. All samples were observed under a fluorescence microscope (AxioImager Z1) or under a confocal microscope (LSM 900) (Zeiss, Jena, Germany) using the AxioVision or Zen program (Zeiss) for image generation, respectively. The ApoTome device (Zeiss) was used for the generation of optical sections using a fluorescence microscope.

##### Labeling of living MDCK and RT4 cells with EryA‐mCherry

Staurosporine‐treated and untreated living cells were incubated with 10 μm EryA‐mCherry for 10 min. After washing with phosphate‐buffered saline (PBS), the cells were fixed with 4% formaldehyde for 15 min, washed with PBS, and mounted in mounting medium Fluoroshield with 4',6‐diamidino‐2‐phenylindole (DAPI, Abcam, ab104139) for nuclei staining.

##### Double labeling of MDCK cells with EryA‐mCherry and anti‐Cas3 antibodies or anti‐CL antibodies

Double labeling with EryA‐mCherry and the anti‐Cas3 antibodies was performed by first applying 10 μm EryA‐mCherry on living STS‐treated and untreated cells for 10 min at 37 °C. After this treatment, the cells were fixed with 4% formaldehyde for 15 min, washed with PBS and then treated for 30 min at 37 °C with the blocking/permeabilization buffer (0.5% BSA, 0.02% sodium azide, 0.1% saponin, 0.1% gelatine and 50 mm NH_4_Cl, in PBS). The EryA‐mCherry labeled cells were afterward incubated with primary anti‐Cas3 antibodies (1 : 50, Abcam, ab2302) or with primary anti‐CL antibodies (1 : 100, USBiological, C1375) overnight at 4 °C, following incubation with goat anti‐rabbit AlexaFluor 488 (A11008, Thermo Fisher Scientific, 1 : 400) or with goat anti‐human AlexaFluor 488 (A11013, Thermo Fisher Scientific, 1 : 400) secondary antibodies for 45 min at 37 °C, respectively. After washing with PBS, the cells were mounted in mounting medium with DAPI.

##### Double labeling of MDCK cells with EryA‐mCherry and annexin V‐FITC

Double labeling with EryA‐mCherry and annexin V‐FITC was performed by applying the mixture of EryA‐mCherry (10 μm) and annexin V‐FITC (final concentration of 0.25–0.5 μg·mL^−1^) on living cells, which were STS‐treated or were left untreated. EryA‐mCherry and annexin‐FITC were incubated with cells for 10 min at 37 °C and 5% CO_2_. The cells were then washed with PBS and mounted in mounting medium with DAPI for nuclei staining. Images from three independent double labelings of EryA‐mCherry and annexin V‐FITC were acquired under the same imaging set‐ups (exposure times, optical section height, magnification). For the quantification of colocalization, the Pearson's correlation coefficient was calculated from the raw optical sections using the Colocalization module (Zen, Jena, Zeiss). Then, Pearson's coefficients were averaged, and a standard error of the mean was calculated.

##### Double labeling of MDCK cells with EryA‐mCherry and DSA‐FITC

Double labeling with EryA‐mCherry and lectin DSA‐FITC was performed by first applying 10 μm EryA‐mCherry on living STS‐treated MDCK cells for 10 min at 37 °C. After this treatment, the cells were fixed with 4% formaldehyde for 15 min and washed with PBS. The EryA‐mCherry labeled cells were afterward incubated with DSA‐FITC for 15 min at 37 °C. After washing with PBS, the cells were embedded in mounting medium with DAPI.

##### Double labeling of MDCK cells with MitoTracker and anti‐CL

Double labeling with MitoTracker Orange CMTMRos and anti‐CL antibodies was performed by first incubating living, untreated cells with 0.1 μm MitoTracker Orange CMTMRos for 30 min at 37 °C, following the manufacturer's instructions. After this, the cells were washed with PBS and then treated for 15 min at 37 °C with the blocking/permeabilization buffer (0.5% BSA, 0.02% sodium azide, 0.1% saponin, 0.1% gelatine, and 50 mm NH_4_Cl in PBS). After washing with PBS, the cells were fixed with 4% formaldehyde for 15 min and washed with PBS. Cells were then incubated with primary anti‐CL antibodies (1 : 100, USBiological, C1375) overnight at 4 °C. The cells were washed with PBS and incubated with goat anti‐human AlexaFluor 488 (A11013, Thermo Fisher Scientific, 1 : 400) antibodies for 45 min at 37 °C. After washing with PBS, the cells were embedded in mounting medium with DAPI.

## Conflict of interest

The authors declare no conflict of interest.

## Author contributions

LŽ, TB, JK, VL, AP, MB, and LLP conducted the experiments; GB, NPU, MS, PV, NR, and KS designed the experiments and wrote the paper. All authors reviewed the manuscript.

## Supporting information


**Fig. S1.** Comparison of EryA‐mCherry circular dichroism (CD) spectra in the absence and presence of large unilamellar lipid vesicles (LUVs) containing cardiolipin (CL).
**Fig. S2.** Permeabilization of small unilamellar lipid vesicles (SUVs) by EryA variants in concert with PlyB at various protein concentrations (as indicated).
**Fig. S3.** EryA‐mCherry labeling in control and staurosporine (STS)‐treated MDCK cells.
**Fig. S4.** EryA‐mCherry labeling in staurosporine (STS) and UV‐treated RT4 cells and MDCK cells.

## Data Availability

The data supporting the findings of this study are available within the manuscript and its Supporting information. Requests for additional information can be directed to the corresponding authors.
