# Supplementary material for: Sensing of cardiolipin exposure on plasma membranes of apoptotic cells by EryA‐mCherry protein
Source: FEBS J. 2025 Oct 23;293(4):1150–67. doi: 10.1111/febs.70290 (PMC12914760; doi:10.1111/febs.70290)
Supplement: Supplementary file 1 — Fig. S1. Comparison of EryA‐mCherry circular dichroism (CD) spectra in the absence and presence of large unilamellar lipid vesicles (LUVs) containing cardiolipin (CL). Fig. S2. Permeabilization of small unilamellar lipid vesicles (SUVs) by EryA variants in concert with PlyB at various protein concentrations (as indicated). Fig. S3. EryA‐mCherry labeling in control and staurosporine (STS)‐treated MDCK cells. Fig. S4. EryA‐mCherry labeling in staurosporine (STS) and UV‐treated RT4 cells and MDCK cells. [file FEBS-293-1150-s001.docx]

**Supplementary data**

**Sensing of cardiolipin exposure on plasma membranes of apoptotic cells by EryA-mCherry protein**

Luka Žeželj^1^, Tadeja Bele^1^, Anastasija Panevska^1^, Gregor Bajc^1^, Jan Kejžar^2^, Miha Bahun^2^, Nataša Poklar Ulrih^2^, Valentina Levak^3,5^, Matej Skočaj^1^, Larisa Lara Popošek^1^, Peter Veranič^4^, Nataša Resnik^4,^*, Kristina Sepčić^1,^*

^1^Department of Biology, Biotechnical Faculty, University of Ljubljana, Jamnikarjeva 101, 1000 Ljubljana, Slovenia

^2^Department of Food Science and Technology, Biotechnical Faculty, University of Ljubljana, Jamnikarjeva 101, 1000 Ljubljana, Slovenia

^3^National Institute of Biology, Večna pot 121, 1000 Ljubljana, Slovenia

^4^Institute of Cell Biology, Faculty of Medicine, University of Ljubljana, Vrazov trg 2, 1000 Ljubljana, Slovenia

^5^Jožef Stefan International Postgraduate School, Jamova 39, 1000 Ljubljana, Slovenia


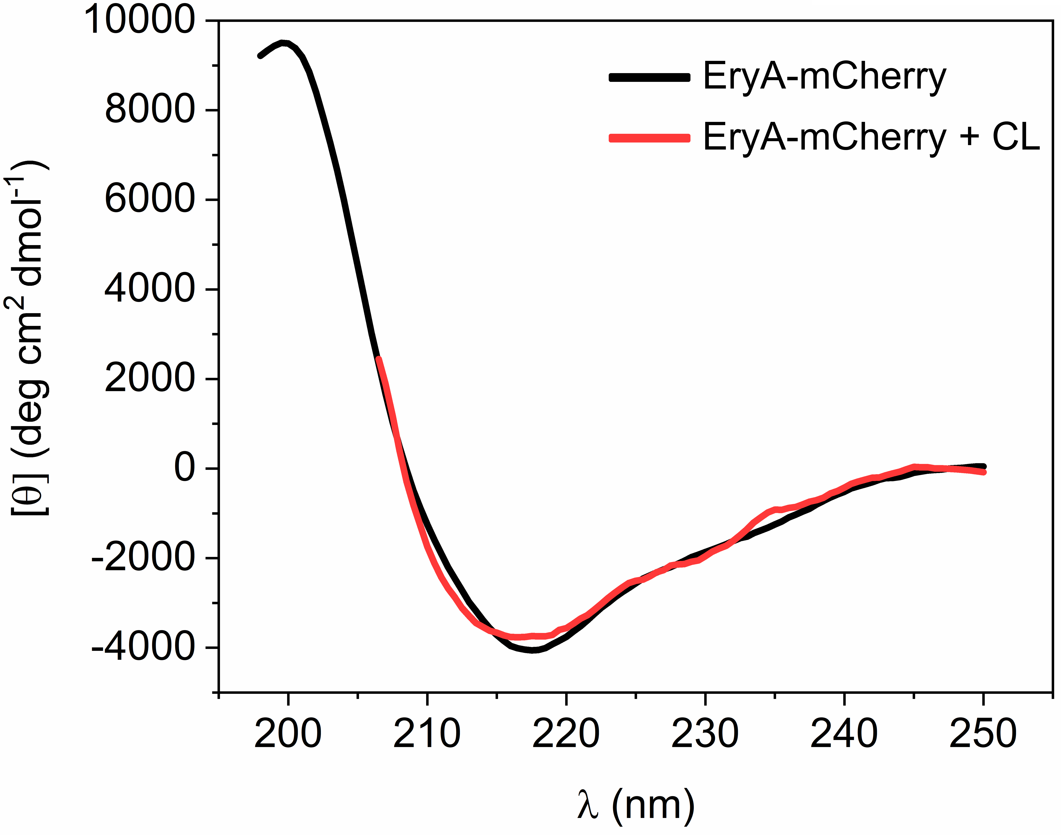


**Figure S1. Comparison of EryA-mCherry circular dichroism (CD) spectra in the absence and presence of large unilamellar lipid vesicles (LUVs) containing cardiolipin (CL).** Black line, CD spectrum of the free EryA-mCherry; red line, CD spectrum of EryA-mCherry in the presence of LUVs composed of equimolar ratios of POPC, cholesterol and CL. Spectra were measured at 25 °C in a quartz cuvette with 1 mm optical length, using a scanning rate of 20 nm/min and bandwidth of 1 nm, with three accumulations per sample. Spectrum of the sample containing the lipids could not be recorded at wavelengths below 206 nm due to high signal noise. Concentrations of EryA-mCherry and lipids in the samples were 0.2 mg/mL and 2.0 mg/mL, respectively. The same buffer (20 mM Tris-HCl (pH 7.4), 140 mM NaCl) was used in all measurements. POPC, 1-palmitoyl-2-oleoyl-sn-glycero-3-phosphocholine.


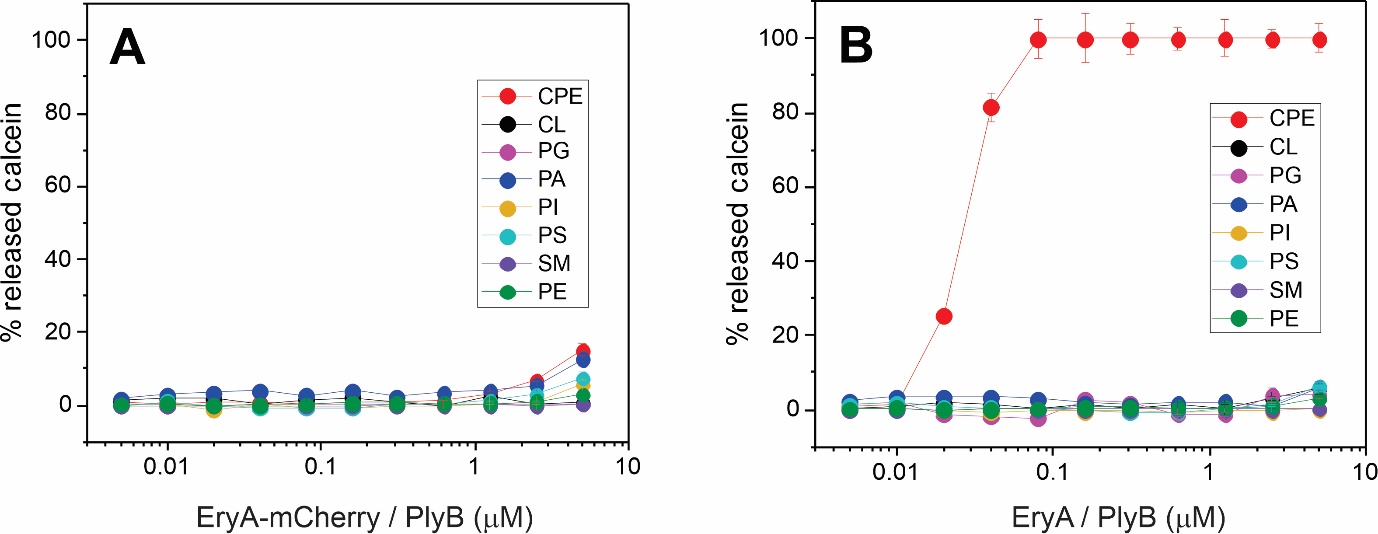


**Figure S2.** **Permeabilization of small unilamellar lipid vesicles (SUVs) by EryA variants in concert with PlyB at various protein concentrations (as indicated).** (A) Permebilization of SUVs by EryA-mCherry/PlyB. (B) Permebilization of SUVs by EryA/PlyB. Lipid vesicles were composed of equimolar ratios of POPC, cholesterol and the third lipid component, as indicated on the graph. EryA-mCherry/PlyB and EryA/PlyB molar ratio, 12.5/1. Data points represent the mean value and SE of three independent measurements. CPE, ceramide phosphoethanolamine; SM, sphingomyelin; CL, cardiolipin; PA, phosphatidic acid; PG, phosphatidylglycerol; PE, phosphatidylethanolamine; PI, phosphatidylinositol; PS, phosphatidylserine; POPC, 1-palmitoyl-2-oleoyl-*sn*-glycero-3-phosphocholine.


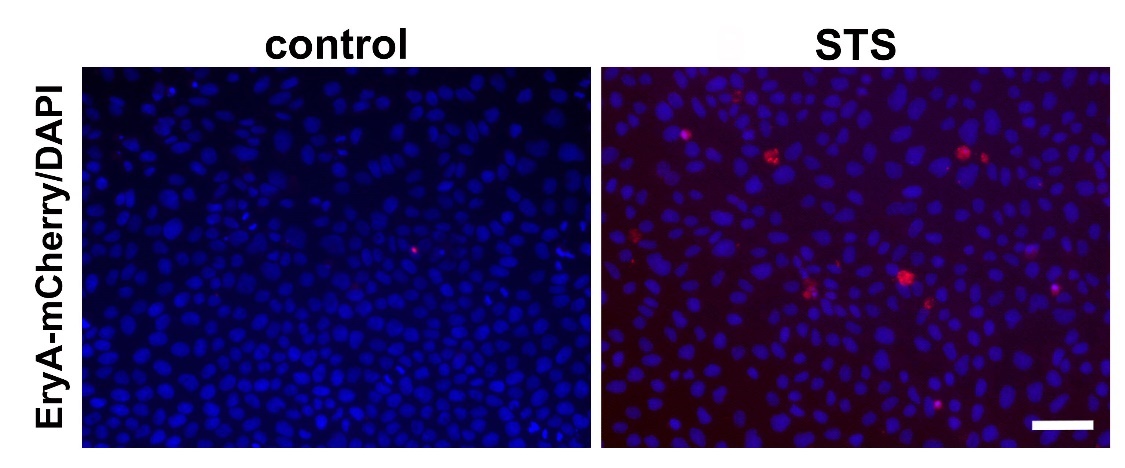


**Figure S3. EryA-mCherry labelling in control and staurosporine (STS)-treated MDCK cells.** Control MDCK cells are not labelled with EryA-mCherry, whereas after STS treatment, cells with evident EryA-mCherry labelling are present. Scale bar: 50 µm.


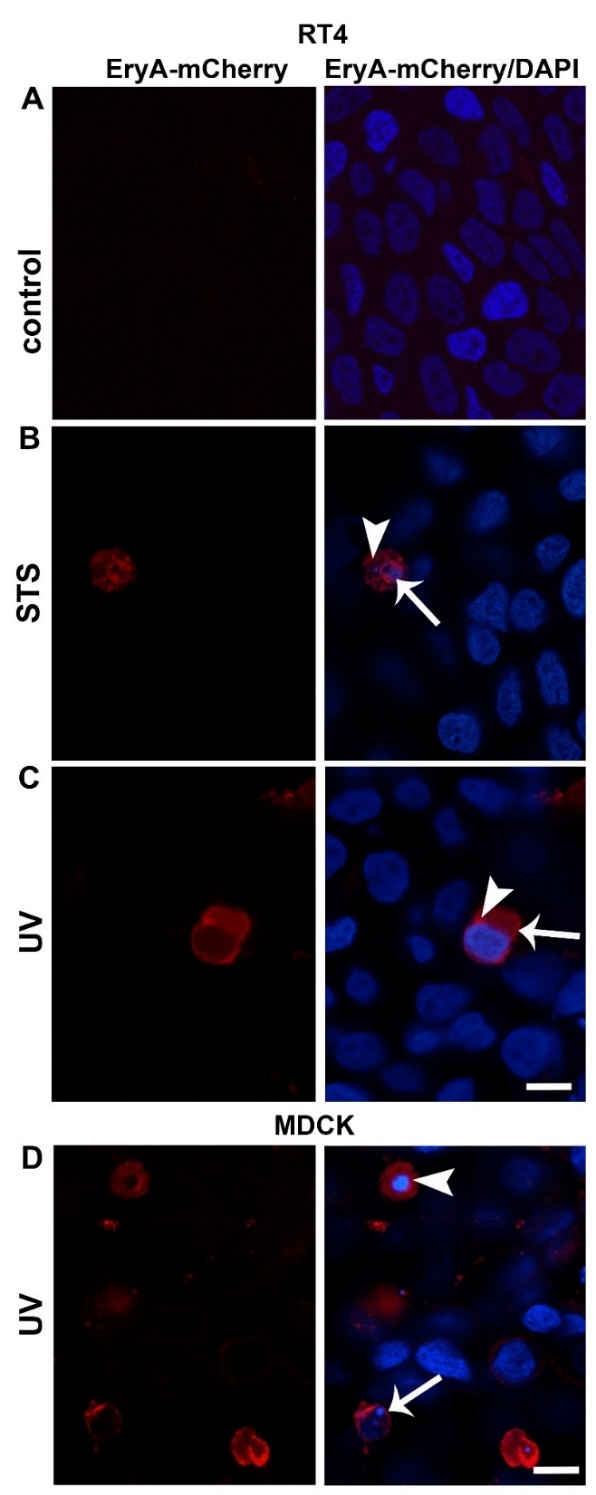


**Figure S4.** **EryA-mCherry labelling in staurosporine (STS) and UV-treated RT4 cells and MDCK cells.** Untreated RT4 cells (**A**), RT4 cells treated with 2 μM STS for 1 hour (**B**) and RT4 cells (**C**) and MDCK cells (**D**) exposed to UV irradiation for 1 min, were labelled with 10 µM EryA-mCherry as described in the Methods. Optical sectioning shows that EryA-mCherry labels round structures, membrane of blebs (arrows) and cytoplasm (arrowhead) in STS- and in UV-induced apoptotic RT4 cells and MDCK cells. Scale bar: 10 µm. Representative fluorescence images of cells labelled with EryA–mCherry, taken from at least three independent experiments, are shown. At least 200 cells per condition were analysed across randomly selected fields (n = 200 cells).
